# Tracking Emergence and Spread of SARS-CoV-2 Omicron Variant in Large and Small Communities by Wastewater Monitoring in Alberta, Canada

**DOI:** 10.3201/eid2809.220476

**Published:** 2022-09

**Authors:** Casey R.J. Hubert, Nicole Acosta, Barbara J.M. Waddell, Maria E. Hasing, Yuanyuan Qiu, Meghan Fuzzen, Nathanael B.J. Harper, María A. Bautista, Tiejun Gao, Chloe Papparis, Jenn Van Doorn, Kristine Du, Kevin Xiang, Leslie Chan, Laura Vivas, Puja Pradhan, Janine McCalder, Kashtin Low, Whitney E. England, Darina Kuzma, John Conly, M. Cathryn Ryan, Gopal Achari, Jia Hu, Jason L. Cabaj, Chris Sikora, Larry Svenson, Nathan Zelyas, Mark Servos, Jon Meddings, Steve E. Hrudey, Kevin Frankowski, Michael D. Parkins, Xiaoli (Lilly) Pang, Bonita E. Lee

**Affiliations:** University of Calgary, Calgary, Alberta, Canada (C.R.J. Hubert, N. Acosta, B.J.M. Waddell, M.A. Bautista, C. Papparis, J. Van Doorn, K. Du, K. Xiang, L. Chan, L. Vivas, P. Pradhan, J. McCalder, K. Low, W.E. England, D. Kuzma, J. Conly, M.C. Ryan, G. Achari, J. Hu, J.L. Cabaj, J. Meddings, K. Frankowski, M.D. Parkins);; University of Alberta, Edmonton, Alberta, Canada (M.E. Hasing, Y. Qiu, T. Gao, C. Sikora, N. Zelyas, S.E. Hrudey, X.[L.] Pang, B.E. Lee); Alberta Health Services, Edmonton (Y. Qiu, C. Sikora, N. Zelyas, X.[L.] Pang);; Alberta Health Services, Calgary (J. Conly, J.L. Cabaj, M.D. Parkins);; University of Waterloo, Waterloo, Ontario, Canada (M. Fuzzen, N.B.J. Harper, M. Servos);; Alberta Health, Government of Alberta, Edmonton (L. Svenson)

**Keywords:** COVID-19, Omicron variant, wastewater, surveillance, pathogens, SARS-CoV-2, public health, communities, variants of concern, coronavirus disease, severe acute respiratory syndrome coronavirus 2, viruses, respiratory infections, zoonoses

## Abstract

Wastewater monitoring of SARS-CoV-2 enables early detection and monitoring of the COVID-19 disease burden in communities and can track specific variants of concern. We determined proportions of the Omicron and Delta variants across 30 municipalities covering >75% of the province of Alberta (population 4.5 million), Canada, during November 2021–January 2022. Larger cities Calgary and Edmonton exhibited more rapid emergence of Omicron than did smaller and more remote municipalities. Notable exceptions were Banff, a small international resort town, and Fort McMurray, a medium-sized northern community that has many workers who fly in and out regularly. The integrated wastewater signal revealed that the Omicron variant represented close to 100% of SARS-CoV-2 burden by late December, before the peak in newly diagnosed clinical cases throughout Alberta in mid-January. These findings demonstrate that wastewater monitoring offers early and reliable population-level results for establishing the extent and spread of SARS-CoV-2 variants.

The COVID-19 pandemic has led to rapid scientific progress in wastewater-based surveillance of community infections. Measuring levels of RNA from SARS-CoV-2 in sewage samples began to be used as a complementary surveillance tool early in the pandemic, resulting in hundreds of wastewater COVID-19 monitoring groups and online dashboards around the world, including in Alberta (https://covid-tracker.chi-csm.ca), a jurisdiction of 4.5 million persons in western Canada. This strategy is premised on the fecal shedding of SARS-CoV-2 by infected persons (*1*,*2*) and modifies quantitative reverse transcription PCR (qRT-PCR) workflows used for diagnosing patients to quantify viral RNA in sewage sampled at wastewater treatment plants or other nodes within the sewer network (*3*–*5*) at regular intervals. Teams in Alberta and elsewhere demonstrated during pandemic waves that wastewater is a leading indicator of COVID-19; results typically precede clinically diagnosed cases by 4–6 days (*6**–**9*). Sampling, testing, and rapidly reporting wastewater virus RNA levels provides early warning of the populationwide disease burden to policy makers, health officials, and the public, enabling evidence-based decision making for preparedness and disease control.

On November 24, 2021, South Africa first reported the emergence of a novel SARS-CoV-2 variant associated with rapid community transmission in the Gauteng province (*10*). By November 26, the World Health Organization had labeled Omicron as a new variant of concern (VOC). Omicron was subsequently rapidly identified in countries around the world, including in Canada, where cases were detected in inbound international travelers. The first case of Omicron from clinical specimen testing in Alberta was confirmed on November 30. By December and into January 2022, the virus had spread rapidly throughout large and smaller communities, prompting reintroduction of public health restrictions (*11*,*12*).

Wastewater testing can differentiate changes in disease burden caused by different VOCs in communities (*13*). As soon as viral genomes of VOCs become available within the international scientific community (e.g., by GISAID, https://www.gisaid.org) (*14*), variant-specific PCR primers and probes can be developed and deployed on regularly collected wastewater samples to learn more about the dynamics of community disease burden caused by VOCs (*15*). Although sequencing viral genomes from wastewater is technically feasible, either through targeted amplicon tiling protocols (*16*,*17*) or shotgun metagenomics (*18*,*19*), a rapid and cost-effective alternative is targeted qRT-PCR screening of RNA extracted from wastewater to provide accurate data on VOCs in near-real time (*20*).

For COVID-19 monitoring in Alberta, wastewater has been sampled, processed, and analyzed in university laboratories in Calgary and Edmonton and the results reported to health officials and online to the public, typically 2 days after sample collection. In this study, we used variant-specific PCR assays to assess the emergence and temporal change in prevalence of the Omicron and Delta variants in Alberta by monitoring wastewater in 30 municipalities, ranging from small towns (population <10,000) to large cities (population >1 million), up to 3 times per week. This approach covered >75% of Alberta’s population of 4.5 million and demonstrated changes in COVID-19 burden associated with emergence of the new Omicron variant from late November 2021 through mid-January 2022.

## Methods

**Figure 1:**
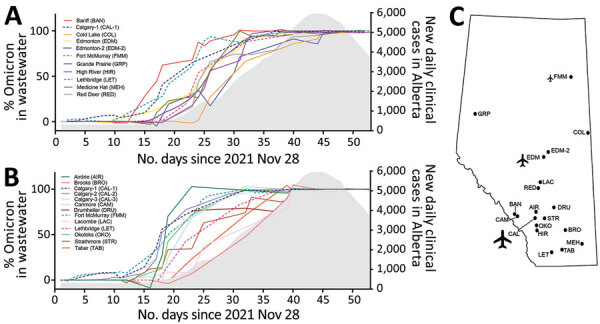
Spread of SARS-CoV-2 Omicron variant in community wastewater samples, Alberta, Canada, November 2021–January 2022. A, B) Percentage of Omicron RNA detected in community wastewater samples (data lines) compared with the 7-day rolling average of new clinical cases reported in Alberta (gray shading). RNA was assessed by using quantitative reverse transcription PCR assays for specific variants following sample processing using ultrafiltration (A) or affinity columns (B). Lines of best fit plotted with second order smoothing are shown for different wastewater treatment plants, including 3 that had samples processed using both ultrafiltration and affinity columns for comparison (Calgary-1, Fort McMurray, and Lethbridge; for details of this comparison, see Appendix Figure 1]). Monitoring began on November 28, 2021, and lasted for 53 days (plotted as consecutive days on the x-axes). The 7-day rolling average of new cases increased after the Omicron variant was predominant in municipal wastewater from 30 communities sampled. C) Locations of 21 treatment plants (Appendix Table) serving communities throughout the province. Abbreviations are as shown in panels A and B. Calgary and Edmonton are served by 3 and 2 treatment plants, respectively, and some individual treatment plants also serve multiple municipalities (e.g., Edmonton-2 serves >10 others; Red Deer serves 3 others; Calgary’s treatment plants serve 3 others).

Wastewater was collected from municipal treatment plants across the province as 24-hour composite samples up to 3 times per week. We isolated RNA from wastewater by using either ultrafiltration followed by RNA extraction (*5*), which was used to process 233 samples, or affinity binding columns that purify nucleic acids directly (*21*), used to process 209 samples (Figure 1). We applied the same method consistently at a given sampling site throughout the entire study period. We processed wastewater samples from 3 geographically disparate treatment plants in Calgary, Fort McMurray, and Lethbridge, comprising 11% of all samples in the study, by using both methods for comparison and revealed no significant difference (Mann-Whitney test: p = 0.46 [Calgary], p = 0.39 [Fort McMurray], and p = 0.59 [Lethbridge]) (Appendix Figure 1).

RNA quantification by qRT-PCR incorporated a newly designed set of assays that selectively amplify the BA.1 Omicron variant or the B.1.617.2 Delta variant by targeting mutations R203K/G204R and R203M in the N200 region of the nucleocapsid gene (M. Fuzzen et al., unpub. data, https://www.medrxiv.org/content/10.1101/2022.04.12.22273761v1). The R203K/G204R mutation in the BA.1 Omicron variant is also present in the B.1.1.7 Alpha variant. Clinical screening of cases indicated that the Alpha variant was no longer detected in Alberta as of July 2021 (*22*). We confirmed this finding by screening the wastewater samples from this study by using a separate assay that targets a D3L mutation in the nucleocapsid gene of the Alpha variant (*23*). We quantified total SARS-CoV-2 levels separately with widely used universal assays targeting the N1 and N2 regions of the nucleocapsid gene in the wild-type virus (*3*,*4*) and all other VOCs identified to date. We triplexed N200 assays for Omicron, Delta, and total SARS-CoV-2 together so we could estimate an Omicron-to-Delta ratio in each wastewater sample using the Omicron signal (R203K-G204R assay) and the Delta signal (R203M assay) (M. Fuzzen et al., unpub. data). This technique enabled us to track the emergence and prevalence of Omicron at the population level throughout the province.

Wastewater sampling and sample processing followed by identifying and quantifying SARS-CoV-2 is intrinsically more complicated than conducting the same PCR strategy on clinical samples (i.e., nasopharyngeal swabs). Directly comparing results between different treatment plants is not normally recommended because of intrinsic heterogeneities (e.g., physiochemical differences manifesting different PCR inhibition potential; different proportions of urban, industrial, and agricultural inputs to urban wastewater; different flow rates and distances affecting signal degradation) (*24*). Other factors, such as population movement between sewershed catchments, can also influence results. These limitations apply to total SARS-CoV-2 quantification and have led to evaluating different population normalization markers in wastewater sample analysis (*25**,**26*). However, the approach presented in this study for determining Omicron-to-Delta ratios within the same multiplex qRT-PCR reaction overcomes these issues, because RNA genomes derived from either variant are expected to react similarly to these factors. In this regard, we confirmed that subsets of samples with 100% Omicron showed good correlation between the R203K-G204R (Omicron) and N1 (total SARS-CoV-2) qRT-PCRs (Appendix Figure 1). We collected daily numbers of new cases of COVID-19 clinically diagnosed across the province by positive PCR test from the Data Analytics branch of Alberta Health Services and reported them using a 7-day rolling average (Figure 1).

## Results

Omicron variant SARS-CoV-2 was first detected in Alberta community wastewater during late November and early December (corresponding to the displacement of the Delta variant) (Figure 1; Appendix Table). In Calgary, 4 consecutive samples collected during December 5–9 revealed the sustained presence of 3%–9% Omicron (compared with >90% Delta) among infected persons contributing to the sewershed in this cosmopolitan city of 1.3 million. Omicron was first detected in wastewater in the capital city of Edmonton (population 1.1 million) on December 10 (15% Omicron, 85% Delta). The rate of increase of Omicron in the international resort town of Banff was higher than in larger cities such as Calgary and Edmonton (Figure 1, panel A), and Omicron surpassed 80% in samples taken 3 times a week during December 20–23 (Appendix Table). By that time, Calgary and Edmonton had just passed 50% Omicron, and the proportion of Omicron infections was growing in smaller bedroom communities adjacent to these 2 large urban centers (e.g., Okotoks, High River, Strathmore, and especially Airdrie, which are all <70 km from Calgary) (Figure 1; Appendix Figure 2). Communities that experienced the most delayed emergence of Omicron were smaller and more remote; Brooks (population 14,451, 190 km from Calgary) and Taber (population 19,070, 263 km from Calgary) did not reach high proportions of Omicron until late December (Appendix Table).

**Figure 2:**
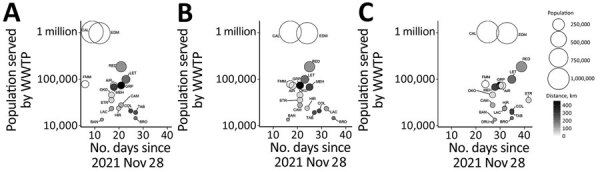
Number of days required for the SARS-CoV-2 Omicron-to-Delta variant ratio to pass thresholds of 10% (A), 50% (B), and 90% (C) of community COVID-19 burden, Alberta, Canada, November 2021–January 2022. General trends of Omicron emergence are shown as a function of decreasing population size and distance from the nearest airport in Calgary, Edmonton, or Fort McMurray. Bubble plots only include data from Calgary-1 and Edmonton-1 wastewater treatment plants (the largest plant from each city), scaled to the population of the corresponding sewershed subcatchment in those cities. AIR, Airdrie; BAN, Banff; BRO, Brooks; CAL, Calgary; CAM, Canmore; COL, Cold Lake; DRU, Drumheller; EDM, Edmonton; FMM, Fort McMurray; GRP, Grande Prairie; HIR, High River; LAC, Lacombe; LET, Lethbridge; MEH, Medicine Hat; OKO, Okotoks; RED, Red Deer; STR, Strathmore; TAB, Taber; WWTP, wastewater treatment plant.

The general trend demonstrated by this analysis of objective wastewater evidence is that large cities experienced the emergence of a newly introduced virus before smaller centers farther away from cities, but with notable exceptions. Banff, located 127 kilometers west of Calgary, experienced a more rapid onset of Omicron infection than anywhere else in the province despite its resident population (13,427 persons) being <1% of the Calgary population and the smallest among monitored communities (Appendix Figure 2, panel B). Banff is an international resort community in Banff National Park, Canada’s busiest national park, which attracts >4 million visitors annually from around the world (*27*). Early detection of Omicron in Banff might correspond to attracting tourists at the onset of the ski season in November and December. Of note, the nearby and slightly larger mountain town of Canmore (population 27,664), located 105 kilometers west of Calgary (22 km east of Banff and outside the national park), experienced a much later emergence of Omicron infections. This delay is likely related to Canmore hosting fewer international tourists than Banff and featuring less high-density dormitory-style housing, where much of the worker population supporting Banff's tourism industry resides.

More remote communities located a greater distance away from Alberta’s large international airports exhibited later emergence of the Omicron variant (Figure 1; Appendix Figure 2). The Calgary International Airport serves 16 million travelers per year with direct flights arriving from 15 countries (*28*), compared with 8 million travelers and 6 countries for Edmonton International Airport (*29*). Plotting Omicron dynamics in Alberta municipalities as a function of distance from Calgary (Appendix Figure 2, panel C) suggests a link to international travel whereby incoming travelers introduce a new virus into a large densely populated urban center, enabling its spread. International travel in and out of Alberta increased sharply in November and December; 22,700 passengers came through Alberta in November and 28,800 in December, compared with only 8,400 travelers during the first 10 months of 2021 combined (*30*). 

Fort McMurray offers an interesting example in relation to domestic air travel. Despite being a remote, relatively small (population 79,205) northern community farther from Calgary than any other municipality sampled in this study, Fort McMurray exhibited an Omicron emergence comparable to the rapid onset in Calgary. Fort McMurray has one of the busiest airports in Canada to accommodate shift workers commuting from across the country to work in the oil sands industry (*31*). This high level of contact with other parts of Canada is likely to result in rapid introduction of an emerging virus such as the Omicron variant. Workers traveling to Fort McMurray from other provinces or from major urban centers in Alberta likely contributed to accelerated Omicron emergence relative to other smaller or remotely situated Alberta municipalities.

The rapid emergence of Omicron in Calgary, Edmonton, Banff, and Fort McMurray is especially evident when this variant comprised lower proportions of the SARS-CoV-2 community burden. The time required for these 4 communities to surpass 10% Omicron was on average (+ SEM) 10.9 + 2.0 days faster than the other communities, highlighting the significantly earlier emergence of Omicron in these locations (p<0.0001 by unpaired t-test) (Figure 2, panel A). These significant differences are maintained by using Omicron cut-offs of 50% (6.3 + 2.7 days faster; p = 0.0331), and the trend is similar at 90% (5.0 + 2.6 days faster; p = 0.0686) (Figure 2, panels B and C). Later emergence of Omicron infections in the less populated outlying communities of Taber, Cold Lake, Lacombe, and Brooks is clearly evident using the 50% cutoff (Figure 2, panel B). In these 4 locations, Omicron surpassed Delta on average 9.8 + 1.8 days later than the other communities, highlighting the significantly slower emergence of infection in these smaller, more remote settings (p<0.0001). Some evidence suggests lower adherence to COVID-19 public health interventions in rural settings than in urban settings, including in Alberta (*32*,*33*), but less densely populated remote areas can experience slower spread of SARS-CoV-2 (*34*,*35*) because of less frequent interaction events, which could potentially contribute to the patterns we report in this study.

## Discussion

Wastewater results demonstrate that the emergence of Omicron was the driver of clinical cases increasing in December and January during Alberta’s fifth wave (Figure 1, panels A, B). During this time, COVID-19 public health surveillance shifted to much more focused PCR testing that prioritized essential workers, patients at risk for severe illness and eligible for early treatment, and patients in emergency departments with more serious illness (*36*). This shift resulted in PCR testing dramatically underestimating total disease burden in the population relative to earlier waves. Reported clinical cases still show a steep increase after the emergence and propagation of Omicron revealed by wastewater testing (Figure 1, panels A, B). These dynamics mirror the shift from Delta to Omicron in Alberta, confirmed clinically by screening subsets of samples using PCR for VOCs and genome sequencing, which revealed levels of Omicron to be >50% by December 16 and >95% by December 28 (*22*). This finding demonstrates that wastewater surveillance reliably provides information vital to public health officials.

VOC information derived from viral genome sequencing of clinical samples in Alberta is nonrandom (which is also the case in many other jurisdictions), placing emphasis on clinical cases of interest (e.g., outbreaks, hospitalizations) or incoming international travelers (*37*). Similarly, clinical PCR testing is susceptible to changes in testing policies, capacity limitations, or persons not getting tested (e.g., by personal choice or when infections are asymptomatic) (*38*). Wastewater testing offers an unbiased representation of disease prevalence, capturing all persons and groups contributing to the sewershed. This comprehensive coverage can be achieved for a tiny fraction of the cost of clinical testing on a per capita basis (*39*). In large cities such as Calgary and Edmonton, which have >1 million residents (Figure 2; Appendix Figure 1), monitoring wastewater for COVID-19 community burden costs only a few cents per person per year (based on testing 3 times per week in Alberta) and can provide objective information about community infection to public health authorities, policy makers, and the public in near-real time. COVID-19 clinical testing strategies and resources are becoming more targeted with jurisdictions such as Alberta turning to self-testing and less frequent public reporting. Wastewater monitoring offers an objective population-based surveillance metric of disease burden that continues to deliver real-time information on COVID-19 and could potentially be adapted for other emerging pathogens.

Appendix Additional information about tracking emergence and spread of SARS-CoV-2 Omicron variant in large and small communities by wastewater monitoring in Alberta, Canada
