# Supplementary material for: Tracking Emergence and Spread of SARS-CoV-2 Omicron Variant in Large and Small Communities by Wastewater Monitoring in Alberta, Canada
Source: Emerg Infect Dis. 2022 Sep;28(9):1770–6. doi: 10.3201/eid2809.220476 (PMC9423933; doi:10.3201/eid2809.220476)
Supplement: Appendix — Additional information about tracking emergence and spread of SARS-CoV-2 Omicron variant in large and small communities by wastewater monitoring in Alberta, Canada [file 22-0476-Techapp-s1.pdf]

Article DOI: https://doi.org/10.3201/eid2809.220476

Tracking Emergence and Spread of SARS-CoV-2 Omicron Variant in Large and Small Communities by Wastewater Monitoring in Alberta, Canada

Appendix

Appendix Table. Chronology of detection of the SARS-CoV-2 Omicron variant in community wastewater, Alberta, Canada*

WWTP or pump station	Date of 1st Omicron detection	≥50% Omicron detected	≥85% Omicron detection	≥99% Omicron detection
Airdrie	Dec 16, 2021	Dec 16, 2021	Dec 16, 2021	Jan 5, 2022
Banff	Dec 12, 2021	Dec 14, 2021	Dec 22, 2021	Dec 27, 2021
Brooks	Dec 29, 2021	Dec 29, 2021	Jan 5, 2022	Jan 5, 2022
Calgary-1†	Dec 1, 2021	Dec 15, 2021	Dec 26, 2021	Dec 29, 2021
Calgary-1‡	Nov 30, 2021	Dec 20, 2021	Dec 28, 2021	Jan 3, 2022
Calgary-2	Dec 7, 2021	Dec 19, 2021	Dec 26, 2021	Jan 4, 2022
Calgary-3	Dec 7, 2021	Dec 19, 2021	Dec 26, 2021	Jan 12, 2022
Canmore	Dec 13, 2021	Dec 20, 2021	Dec 29, 2021	Jan 29, 2022
Cold Lake	Dec 16, 2021	Dec 29, 2021	Jan 5, 2022	Jan 11, 2022
Drumheller	Dec 20, 2021	Dec 20, 2021	Dec 29, 2021	Jan 17, 2022
Edmonton-1	Dec 10, 2021	Dec 22, 2021	Dec 28, 2021	Jan 17, 2022
Edmonton-2	Dec 8, 2021	Dec 22, 2021	Dec 29, 2021	Jan 14, 2022
Fort McMurray†	Dec 1, 2021	Dec 20, 2021	Dec 22, 2021	Jan 5, 2021
Fort McMurray‡	Dec 6, 2021	Dec 15, 2021	Dec 22, 2021	Dec 22, 2021
Grande Prairie§	Dec 20, 2021	Dec 20, 2021	Dec 28, 2021§	Dec 28, 2021§
High River	Dec 20, 2021	Dec 22, 2021	Dec 29, 2021	Dec 30, 2021
Lacombe	Dec 16, 2021	Jan 5, 2022	Jan 5, 2022	Jan 5, 2022
Lethbridge†	Dec 14, 2021	Dec 21, 2021	Dec 29, 2021	Jan 10, 2022
Lethbridge‡	Dec 19, 2021	Dec 28, 2021	Dec 28, 2021	Jan 5, 2022
Medicine Hat	Dec 16, 2021	Dec 23, 2021	Dec 30, 2021	Jan 4, 2022
Okotoks	Dec 13, 2021	Dec 20, 2021	Dec 20, 2021	Jan 6, 2022
Red Deer	Nov 29, 2021	Dec 23, 2021	Jan 4, 2022	Jan 17, 2022
Strathmore	Dec 15, 2021	Dec 20, 2021	Jan 5, 2022	Jan 19, 2022
Taber	Dec 29, 2021	Dec 29, 2021	Dec 29, 2021	Jan 5, 2022

*WWTP, wastewater treatment plant.
†Wastewater samples processed using the ultrafiltration method.
‡Wastewater samples processed using the affinity column method.
§Values dropped below 85% and 99% and then increased again after the indicated dates.

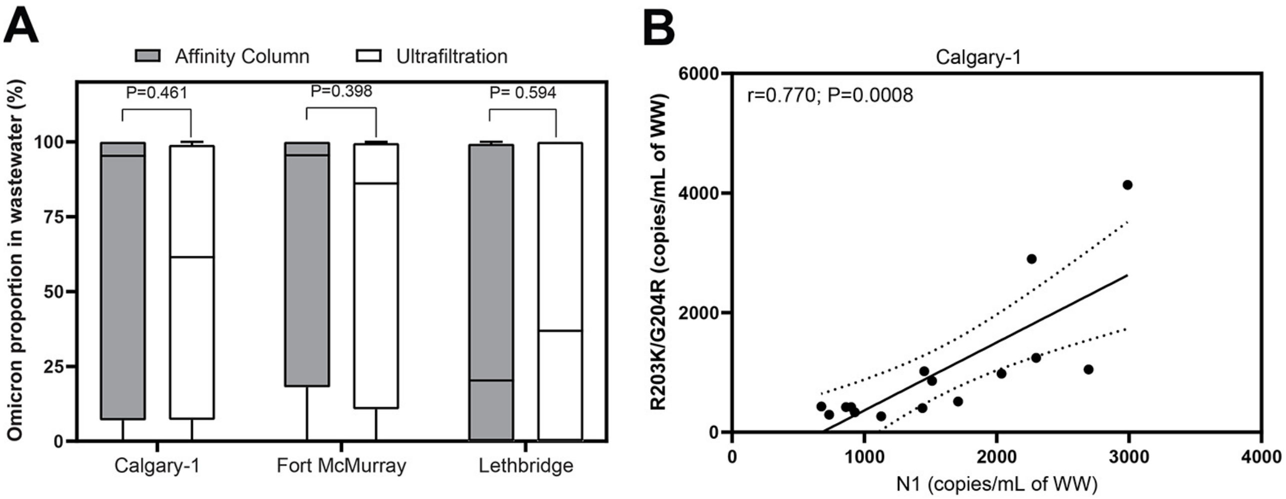

Appendix Figure 1. (A) Wastewater sample processing using affinity columns and ultrafiltration was compared by testing samples from wastewater treatment plants in Calgary-1 (n = 14), Fort McMurray (n = 18), and Lethbridge (n = 15) using both methods. The proportion of Omicron obtained following either processing method was compared using Mann-Whitney tests revealing no significant difference in median values. Median and interquartile ranges are indicated as the middle, top, and bottom lines of each box. Ends of the whiskers mark the lowest and highest ratios determined in each sample series. (B) These sample sets were also used to compare quantitative reverse transcription PCR assay results in samples with 100% Omicron for correlation of the Omicron-specific R203K/G204R assay with the universal N1 gene assay. Pearson correlation is shown for Calgary-1 samples, which was similar to that observed in comparison of Lethbridge (r = 0.608; p = 0.209) and Fort McMurray (r = 0.595; p = 0.0415) samples. WW, wastewater.

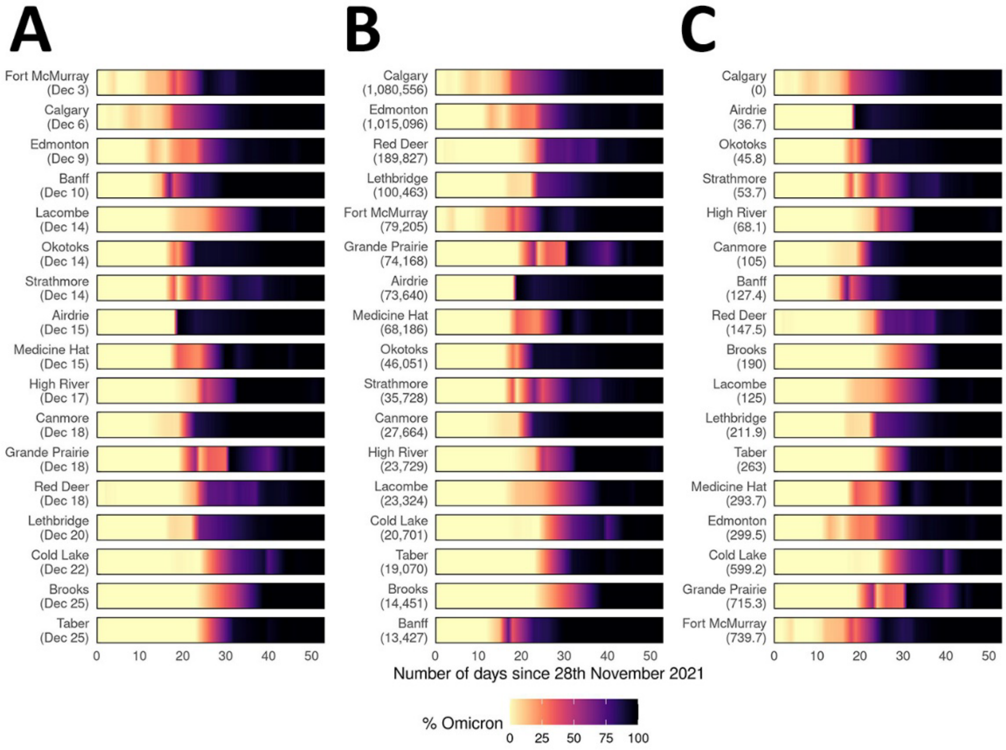

Appendix Figure 2. Proportion of Omicron in Alberta, Canada, municipalities plotted in order of the timing of its emergence to 10% of the overall signal (A). Presenting the communities in order of decreasing population size (B) highlights that Banff (the wastewater catchment with the smallest population) does not follow the predicted trend of later emergence in smaller communities. Presenting the communities in order of increasing distance from the Calgary International Airport (C) highlights that Fort McMurray (the most remote community) does not follow the predicted trend of earlier emergence in communities closer to Calgary. Edmonton and Calgary results shown here are limited to the largest WWTPs in the 2 cities (i.e., Edmonton-1 and Calgary-1 from Figure 1). Timing in panel A corresponds to the midpoint between sampling dates with values below and above 10%. WWTP, wastewater treatment plant.
